# Drug poisoning deaths in the United States, 1999–2012: a statistical adjustment analysis

**DOI:** 10.1186/s12963-016-0071-7

**Published:** 2016-01-15

**Authors:** Christopher J. Ruhm

**Affiliations:** 1 Frank Batten School of Leadership and Public Policy, University of Virginia, Garrett Hall 204, 235 McCormick Road, P.O. Box 400893, Charlottesville, VA 22904-4893 USA; 2 National Bureau of Economic Research, Cambridge, MA USA

**Keywords:** Fatal drug poisonings, Drug poisoning mortality, Drug involvement, Poisoning deaths, Death certificates

## Abstract

**Background:**

Drug poisoning mortality in the US has risen rapidly but the drugs involved are frequently unspecified on death certificates.

**Methods:**

Reported and adjusted proportions of specific drug types involved in fatal drug poisonings were calculated using vital statistics mortality data from 1999 to 2012. The adjusted proportions were those predicted to occur if at least one specific type of drug had been identified on the death certificates of all poisoning fatalities.

**Results:**

Adjusted involvement rates of opioid analgesic mentions in 2012 were 54.3 % (95 % confidence interval [CI]: 53.6 %–55 %), 40.8 % higher than the reported 38.6 % rate. Adjusted rates for all narcotics, other narcotics, sedatives, or psychotropics, and multiple drug use were 81.5 % (95 % CI: 80.9 %–82.2 %), 38.4 % (95 % CI: 37.8 %–39 %), 30 % (95 % CI: 29.4 %–30.7 %), 26 % (95 % CI: 25.4 %–26.6 %) and 42.8 % (95 % CI: 42.1 %–43.5 %) in 2012, compared to reported proportions of 60.7, 27.9, 18.7, 18 and 26.9 %. The adjustments typically had similar or slightly smaller effects on the estimates in 1999, and larger impacts on subcategories of drug types such benzodiazepines and antipsychotic medications. Based on the adjusted proportions, 22,534, 15,933, 12,457, 10,798, and 17,670 drug deaths in 2012 were estimated to involve opioid analgesics, other narcotics, sedatives, psychotropic medications, and drug combinations, compared to death certificate reports of 16,007, 11,567, 7,754, 7,467, and 11,176.

**Conclusions:**

Death certificates substantially understate the involvement of opioid analgesics, sedatives, psychotropics, and drug combinations in fatal drug poisonings. Adjustment procedures that account for cases where only unspecified drugs are reported on death certificates provide more accurate information.

**Electronic supplementary material:**

The online version of this article (doi:10.1186/s12963-016-0071-7) contains supplementary material, which is available to authorized users.

## Background

The poisoning mortality rate roughly tripled over the last three decades, with about 90 % of these fatalities now caused by drugs [1]. At least 80 % of drug fatalities were accidental in 2011 and these are now the leading cause of injury deaths [2]. Increased rates of poisoning deaths are the most important reason for the striking result that the all-cause mortality rates of 45–54 year old non-Hispanic whites rose by around 0.5 % per year between 1999 and 2013 [3]. The involvement of prescription opioid analgesics, such as oxycodone, methadone, and hydrocodone has received considerable attention [1, 4–7]. But drug poisonings are not limited to opioids – sedatives and psychotropic drugs are frequently identified on death certificates, and combination drug use is common [8, 9].

There are several barriers to formulating the most effective policies to deter dangerous use of prescription pharmaceuticals while avoiding the potential substitution to other harmful legal or illegal drugs. Perhaps most important is that we do not currently have reliable information on the specific drugs involved in fatal drug poisonings because no specific drug is identified on almost one-quarter of death certificates. As a result, rates of involvement for specific legal and illegal drugs are underestimated, as is the simultaneous use of drug combinations.

This study provides a more accurate understanding of the nature of drug poisoning deaths by using statistical methods that account for cases where no specific drug is identified on death certificates, to estimate the proportions of specific drug mentions and combination drug use for all types of drug poisoning mortality, as well as separately by manner of death (accidental, intentional or undetermined) and for two age groups (15–59 and ≥60 year olds). Adjusted counts of fatal drug poisonings involving specified drug types are also calculated and contrasted with those based on death certificate reports. Data come from the 1999–2012 Centers for Disease Control and Prevention (CDC) *Multiple Cause of Death (MCOD)* files, described in detail elsewhere [10].

## Methods

The *MCOD* files are based upon death certificates. Each certificate contains a single underlying cause of death, up to 20 additional causes, and demographic data. Information is utilized on cause of death, using four-digit *International Classification of Diseases, Tenth Revision* (ICD-10) codes, place of residence, age, race/ethnicity, gender, year, and weekday of death. The public use files lack geographic identifiers for most years; however, information on the state and county of residence, available under restricted conditions, was obtained and used for this analysis. The Institutional Review Board for the Social and Behavioral Sciences at the University of Virginia reviewed this project and determined that it does not involve the use of human subjects.

The investigation is limited to deaths occurring to US residents (foreign residents dying in the US were excluded). The main analysis begins in 1999 because ICD-9 codes, used prior to that year, are not fully comparable to ICD-10 categories [11]. Poisoning and drug poisoning deaths were defined using ICD-10 underlying cause of death (UCD) codes, where the UCD is the “disease or injury that initiated the chain of morbid events that led directly and inevitably to death” [12]. Poisoning deaths included ICD-10 codes X40-X49, X60-X69, X85-X90 Y10-Y19; Y35.2; *U01(.6-.7); ICD-10 codes for drug poisoning deaths were X40-X44, X60-X64, X85, Y10-Y14; Y35.2; *U01(.6-.7) [13].

In cases of drug poisoning, the death certificate lists one or more drugs involved as immediate or contributory causes of death. These were included separately in the *MCOD* files as ICD-10 “T-codes” and are referred to below as drug mentions. Specific drug categories examined include: narcotics, sedatives, psychotropics, other specified drugs, and unspecified drugs. Important subcategories of drug involvement were also analyzed. Narcotics were decomposed into (prescription) opioid analgesics and other narcotics; opioid analgesics into methadone and other opioid analgesics; other narcotics into heroin, cocaine, and other non-analgesic opioids. Benzodiazepines were sometimes separately broken out as an important subclass of sedatives. Among psychotropic medications, antidepressants, antipsychotics, and stimulants were separately examined. Other specified drugs comprised a wide variety of medications including anesthetics, antiallergic and immunosuppressive drugs, histamine and anti-gastric secretion medications, cardiac drugs, antibiotics and many others; deaths involving these drugs occurred relatively rarely and so were examined as a group. Poisoning by unspecified drugs, medicaments and biologicals (ICD-10 code, T50.9) was important because no specific drug was identified for approximately one-quarter of drug poisoning deaths. Combination drug use was examined through a constructed variable indicating mentions of two or more of the following drug categories: opioid analgesics, other opioids, sedatives, psychotropics, or other specified drugs.

Numbers and percentages of drug poisoning deaths were specified by type of drug, with the shares sometimes referred to as proportions below. Results are reported for 1999 and 2012, the first and last analysis years. Shares were also computed for subsamples of drug poisonings stratified by whether they were classified based on the underlying cause of death as accidental (UCD codes: X40-44), intentional (X60-64), of undetermined intent (Y10-Y14), or resulting from homicide/legal intervention (X85, Y35.2, *U01(.6-.7)). Proportions were also calculated separately for 16–59 and ≥60 year olds. Results are not shown for drug deaths involving persons younger than 16 because these occur too rarely (92 cases in 1999 and 147 in 2012) for adjusted proportions to be reliably estimated. Table 1 shows ICD-10 codes for each drug mention and manner of death category [14].

**Table 1:** Drug involvement in drug poisoning deaths: 1999 and 2012^a^

Drug category/Manner of death	1999		2012	
	Percent	Number	Percent	Number
All drug deaths (X40-44, X60-64, X85, Y10-14, Y35.2, *U01(.6-.7))^b^	100.0	16,849	100.0	41,502
Drug mentions (T-codes)				
Narcotics (40.0-40.9)	58.9	9922	60.7	25,187
Opioid analgesics (40.2-40.4)	23.9	4030	38.6	16,007
*Methadone (40.3)*	*4.7*	*784*	*9.5*	*3932*
*Other opioid analgesics (40.2, 40.4)*	*19.9*	*3360*	*31.4*	*13,012*
Other narcotics (40.0–40.1, 40.5–40.9)	42.4	7137	27.9	11,567
*Heroin (40.1)*	*11.6*	*1960*	*14.3*	*5925*
*Cocaine (40.5)*	*22.7*	*3822*	*10.6*	*4404*
*Miscellaneous (40.0, 40.6*–*40.9)*	*17.4*	*2931*	*7.2*	*3006*
Sedatives (40.2–40.8)	9.9	1662	18.7	7754
*Benzodiazepines (42.4)*	*6.7*	*1135*	*15.7*	*6524*
*Other sedatives (42.0*–*42.3, 42.5*–*42.8)*	*3.9*	*663*	*4.9*	*2048*
Psychotropics (43.0–43.9)	14.6	2466	18.0	7467
*Antidepressants (43.0*–*43.2)*	*10.4*	*1749*	*10.3*	*4259*
*Antipsychotics (43.3*–*43.5)*	*1.9*	*321*	*3.2*	*1333*
*Stimulants (43.6)*	*3.2*	*547*	*6.3*	*2635*
Other specified (36.0–38.9, 41.0, 41.9, 44.0–48.7, 49.0–50.8)	6.9	1171	7.6	3156
Unspecified (50.9)	50.3	8477	49.7	20,612
>1 Major drug class^c^	18.0	3040	26.9	11,176
Manner of death				
Accidental (X40–44)	66.2	11,155	79.9	33,175
Intentional (X60–64)	18.9	3181	13.2	5465
Undetermined intent (Y10–Y14)	14.7	2473	6.7	2782
Homicide (X85, Y35.2, *U01(.6–.7))	0.2	40	0.2	80

^a^Data from the Multiple Cause of Death files

^b^Entries in parentheses refer to ICD-10 X and Y codes for the underlying causes of death used to classify drug poisoning and manner of death, and T codes for drug mentions

^c^Two or more of the drug types: opioid analgesics, other narcotics, sedatives, psychotropics, or other specified drugs

A dichotomous variable was constructed indicating if at least one specific drug was identified on the death certificate, rather than only the unspecified drug category being listed. State-year and county-year averages of this variable were calculated, with the latter denoted as *SPECIFY*. States were classified as “low diagnosis” if mention of a specific drug was provided in fewer than 68.8 % of drug poisoning deaths in both 1999 and 2012 and as “high diagnosis” if this was done on more than 89.6 % of cases in both years (Additional file 1). These thresholds reflected the 25^th^ and 75^th^ percentiles of drug specification rates over the 1999–2012 period. The within-state correlation in this variable across 1999 and 2012 was large but not perfect (*r* = 0.851).

Drug mention and manner of death shares were compared across high and low diagnosis states for 1999 and 2012 to provide a first indication of how reported proportions were affected by the frequent failure to identify any of the specific drugs involved in fatal drug poisonings. Such comparisons will not be fully informative if high and low diagnosis states differ along other dimensions. To control for potential confounding factors, a series of probit models were separately estimated for 1999 and 2012.

Probit is a nonlinear regression model specifically designed for binary dependent variables, such as drug mentions. It is similar to logit (logistic) regression except that the cumulative distribution function of the error term has a standard normal, rather than logistic, distribution [15]. The basic probit model took the form:

1 $$ {Y}_{ijt}=\alpha +\beta SPECIF{Y}_{jt}+\gamma {X}_{ijt}+{\mu}_{ijt}, $$

where *Y*_*ijt*_ was a binary dependent variable indicating if the death for individual *i* in county *j* and year *t* was reported to involve the specified drug type. *SPECIFY*, the explanatory variable of primary interest, measured the county-year drug specification rate. *μ* is a regression error term.

Predictive power of the model was improved by including supplementary covariates (*X*) capturing the effects of characteristics related to the likelihood of the specified drug being involved in the death but not caused by county differences in *SPECIFY*. The main specifications included controls for: gender, two race indicators (black and other nonwhite), being married at the time of death (versus never married, separated/divorced, widowed, or status not reported), four educational categories (less than high school graduate, high school graduate, some college, college graduate, with education not reported as the reference group), eight age categories (≤20, 21–30, 31–40, 41–50, 51–60, 61–70, 71–80, >80, with missing age as the reference group), nine census regions (New England, Mid-Atlantic, East North Central, West North Central, South Atlantic, East South Central, West South Central, Mountain and Pacific), and seven day of the week of death indicators. Education was sometimes reported in years rather than specific thresholds. In these cases, ≤11, 12, 13–15, and ≥16 years were classified as less than high school graduate, high school graduate, some college, and college graduate. Manner of death was not controlled for in the primary specifications, because of evidence provided below that classification varies with county-level drug specification rates and previous research indicating that death certificates sometimes misclassify intentional drug fatalities as being accidental or of undetermined intent [16, 17]. Supplementary models added controls for the manner of death (accidental or intentional, with undetermined intent/homicide as the reference group) allowing for proportions to be compared with and without the inclusion of these supplementary controls.

Predicted values of the dependent variable were calculated for each drug poisoning death and then averaged over all observations to obtain estimated proportions. The average predicted proportion, ${\overline{P}}_{jt},$ for drug type *j* at time *t*, was:

2 $$ {\overline{P}}_{jt}=\frac{1}{n}{\displaystyle {\sum}_{i=1}^n}\Phi \left({\widehat{Y}}_{ijt}\right) = \frac{1}{n}{\displaystyle {\sum}_{i=1}^n}\Phi \left(\widehat{\alpha}+\widehat{\beta} Specif{y}_{jt}+\widehat{\gamma}{X}_{ijt}\right), $$

for Φ(.), the cumulative distribution function of the standard normal distribution. Since these predictions were based on actual values of the explanatory variables, the estimated proportions were expected to be similar to the sample mean values. This was tested for and confirmed for all types of drug mentions in both 1999 and 2012 (Additional file 2).

Next, a second set of predicted values was obtained after setting *SPECIFY* to one for all observations. The average expected value, hereafter referred to as the “adjusted proportion”, ${\tilde{P}}_{jt}$, was estimated as:

3 $$ {\tilde{P}}_{jt}=\frac{1}{n}{\displaystyle {\sum}_{i=1}^n}\varPhi \left(\widehat{\alpha}+\widehat{\beta}+\widehat{\gamma}{X}_{ijt}\right), $$

and indicates the drug involvement rate expected if at least one specific drug type had been identified on all drug poisoning death certificates. Ninety-five percent confidence intervals (95 % CI) were calculated using the delta method.

The predicted number of deaths involving the specified class of drugs, ${\tilde{D}}_{jt}$, was computed as the product of the adjusted proportion and number of drug poisoning deaths in year *t*, *D*_*t*_*,* or:

4 $$ {\tilde{D}}_{jt}={\tilde{P}}_{jt}\times {D}_t, $$

The associated lower (upper) threshold of the 95 % CI was computed as the product of the number of drug fatalities times the lower (upper) value of the adjusted proportion 95 % CI.

Although there is no single agreed-upon measure of goodness-of-fit in probit or logit models (corresponding to R^2^ in a regression framework), a common measure used here is the pseudo-R^2^, defined as 1 − ℒ_*ur*_/ℒ_0_, where ℒ_*ur*_ is the log-likelihood function of the estimated specification and ℒ_0_ is the log-likelihood function of the intercept-only model [18]. Two additional indications of the success of the adjustment procedures were examined. The first compared reported and adjusted shares of exclusive unspecified drug mentions (those where no drug was identified on the death certificate). As mentioned, the reported proportion was approximately 25 % in most years. Completely successful adjustment procedures would reduce this to zero, and estimated shares close to this would provide confidence in the adjustment procedure. The second test was the reverse of the first. Here, adjusted proportions were calculated using the same procedure but assuming that drug types were never specified on the death certificates, by predicting proportions after setting *SPECIFY* to zero as: ${\tilde{P}}_{jt} = \frac{1}{n}{\displaystyle {\sum}_{i=1}^n}\varPhi \left(\widehat{\alpha}+\widehat{\gamma}{X}_{ijt}\right)$. Perfect adjustment would imply exclusive mentions of unspecified drugs in 100 % of drug fatalities. Predicted probabilities can never reach zero or one in a probit model, so that complete adjustment will never be achieved.

Analyses were conducted using STATA Statistical Software: Release 14 [19]. Statistical significance refers to *P* < .05, from two-sided tests.

## Results

### Trends in drug poisoning and specific drug mention rates

**Fig. 1:**
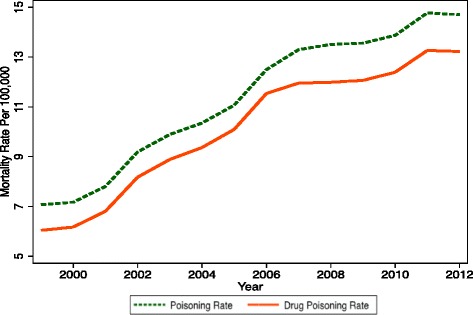
Poisoning and drug poisoning mortality rates

**Fig. 2:**
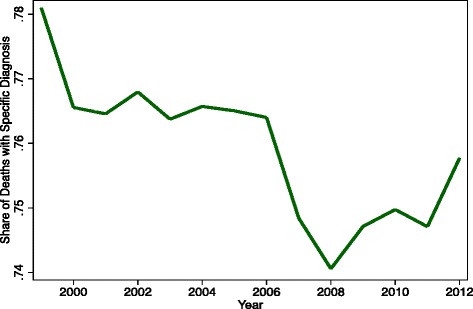
Drug specification rate

Drug deaths rose 146 % between 1999 and 2012, from 16,849 to 41,502. The national population grew by 12.5 % and the total number of deaths by 6.4 % over the same period, so that the (non-age-adjusted) fatal poisoning rate increased by 119 % from 6.04 to 13.22 per 100,000 and from 0.70 % to 1.55 % of all deaths. Poisoning mortality of all types grew 134 % (from 19,741 to 46,150) between 1999 and 2012, and drug fatalities as a share of poisoning deaths increased from 85 % in 1999 to 92 % in 2006, before declining slightly to 90 % in 2012 (Fig. 1). The rise in drug mortality represents an acceleration of a trend that started in the early 1980s. In 1982, 6518 individuals died from drugs, or 2.81 per 100,000 population, and these constituted 58 % of all poisoning fatalities [1]. The drug poisoning death rate grew 4.6 % annually between 1982 and 1999 (from 2.81 to 6.04 per 100,000) versus 6.2 % per year from 1999 to 2012 (from 6.04 to 13.22 per 100,000). One or more specific drugs were mentioned for 78.1, 73.1, and 75.8 % of drug fatalities in 1999, 2008, and 2012 (Fig. 2).

### Death certificate reports

Narcotics were identified in 60.7 % of all 2012 drug fatalities, slightly above 58.9 % in 1999 (Table 1). This reflected a rapid increase in opioid analgesic mentions (from 23.9 % to 38.6 %) and heroin-involved deaths (from 11.6 % to 14.2 %) that more than offset substantial reductions in reported shares of cocaine and miscellaneous narcotics. However, since drug deaths increased dramatically between 1999 and 2012 (from 16,849 to 41,502), the number of drug mentions sometimes increased even while proportions declined. For example, cocaine mentions grew from 3822 to 4404 fatalities while the corresponding share of drug deaths fell from 22.7 % to 10.6 %.

These overall patterns conceal heterogeneity within drug classes. Methadone mentions more than quintupled over the 13 years, while other opioid analgesic involvement less than quadrupled. The 367 % increase in sedative involvement was largely due to the 475 % rise in benzodiazepine mentions, and reported psychotropic drug mentions grew 203 %, led by 382 % and 315 % increases in stimulant and antipsychotic involvement. Mentions of other specified drugs rose 170 %. Also noteworthy was the rise from 8477 to 20,612 in unspecified drug involvement that exceeded opioid analgesic mentions by 29 % in 2012. Drug combinations were reported in 18 % fatal drug poisonings in 1999 and 27 % in 2012.

The classified manner of drug fatalities changed over time, with the share of accidental poisonings rising from 66 % to 80 % between 1999 and 2012, while intentional deaths fell from 19 % to 13 % and those of undetermined intent from 15 % to 7 %; homicides represented a miniscule 0.2 % of drug poisoning fatalities in both years (bottom panel of Table 1).

### Adjusted shares

Death certificates understate most drug involvement proportions because of the frequency of cases where no specific drug was identified. A first indication of this was obtained by comparing death certificate reports in high and low diagnosis states (Table 2). As expected, the reported proportions were almost always greater in the high diagnosis locations. For example, narcotics involvement in 2012 was 78.3 % in high diagnosis states versus 41.1 % in those with low diagnosis rates. Substantial differences were obtained for opioid analgesics, sedatives, psychotropics, and combination drug use, while unspecified drugs were less commonly mentioned, with 36.5 % for high versus 68.4 % for low diagnosis in 2012. One exception is that heroin mentions were more prevalent in low than high diagnosis states in 1999 (but not 2012).

**Table 2:** Drug involvement in drug poisoning deaths in high and low diagnosis states^a^

Drug category/Manner of death	1999		2012	
	Low diagnosis states^b^	High diagnosis states^c^	Low diagnosis states^b^	High diagnosis states^c^
Drug mentions				
Narcotics	35.9	76.1	41.1	78.3
Opioid analgesics	13.4	23.8	24.7	52.1
Other narcotics	25.0	60.7	20.0	36.5
*Heroin*	*10.8*	*7.9*	*12.2*	*18.5*
*Cocaine*	*13.5*	*29.3*	*6.6*	*13.5*
Sedatives	5.6	11.0	10.6	24.8
*Benzodiazepines*	*3.8*	*7.4*	*9.0*	*20.2*
Psychotropics	7.7	17.0	8.5	23.9
*Antidepressants*	*6.1*	*13.2*	*5.9*	*14.7*
*Antipsychotics*	*1.0*	*2.4*	*1.5*	*4.6*
*Stimulants*	*1.0*	*3.2*	*1.9*	*7.2*
Other specified	7.4	6.4	4.9	11.0
Unspecified	66.7	30.7	68.4	36.5
>1 Major drug class^d^	8.1	21.5	15.1	39.8
Drug poisoning rate^e^	5.04	7.00	14.09	13.37
Manner of death				
Accidental	64.1 %	49.6 %	80.6 %	73.5 %
Intentional	19.1 %	16.1 %	10.9 %	13.5 %
Undetermined intent	16.2 %	34.0 %	8.3 %	12.9 %
Homicide	0.6 %	0.3 %	0.2 %	0.1 %

^a^Data from the Multiple Cause of Death files

^b^Low diagnosis states, defined as those with at least one drug specified for fewer than 68.8 % of drug poisoning deaths in both 1999 and 2012, include: Alabama, Idaho, Indiana, Louisiana, Michigan, Mississippi, Montana, Nebraska, North Dakota and Pennsylvania

^c^High diagnosis states, defined as those with at least one drug specified for more than 89.6 % of drug poisoning deaths in both 1999 and 2012 include: Alaska, Maryland, Massachusetts, Nevada, New Hampshire, New York, Oklahoma, Rhode Island, Utah, Virginia and Washington

^d^Two or more of the drug types: opioid analgesics, other narcotics, sedatives, psychotropics, or other specified drugs

^e^Drug poisoning rate is per 100,000 population

This comparison does not account for potential confounders. As evidence that these may matter, drug poisoning rates almost tripled in the low diagnosis states (from 5.0 to 14.1 per 100,000) between 1999 and 2012 compared to less than doubling (from 7.0 to 13.4 per 100,000) in high diagnosis areas. There are also substantial differences in the classified manner of deaths, with accidental fatalities being more common and those with undetermined intent being less frequent in low than in high diagnosis states. To the extent that these represent differences in reporting patterns rather than actual mechanisms of death, errors may be introduced when including the controls for the manner of death when estimating adjusted proportions or when examining differences in shares across reported manners of drug deaths.

Table 3 displays reported and adjusted proportions, as well as percentage differences between the two, for 1999 and 2012. As mentioned, adjusted involvement was estimated from probit models that account for spurious correlation related to differences in age, sex, race/ethnicity, educational attainment, census region, and the weekday of the death, and calculated under the assumption that at least one specific drug was identified for each drug poisoning fatality.

**Table 3:** Reported and adjusted drug involvement^a^

Drug category	% [95 % CI]					
	1999			2012		
	Reported^b^	Adjusted^c^	% Difference^d^	Reported^b^	Adjusted^c^	% Difference^d^
Narcotics	58.9	77.8 [76.9–78.7]	32.2 [30.6–33.7]	60.7	81.5 [80.9–82.2]	34.3 [33.2–35.4]
Opioid analgesics	23.9	31.3 [30.2–32.4]	30.9 [26.2–35.6]	38.6	54.3 [53.6–55.0]	40.8 [39.0–42.5]
Other narcotics	42.4	58.3 [57.2–59.3]	37.6 [35.1–40.1]	27.9	38.4 [37.8–39.0]	37.7 [35.5–40.0]
*Heroin*	*11.6*	*14.1 [13.3*–*14.9]*	*20.9 [14.2*–*27.7]*	*14.3*	*20.0 [19.4*–*20.5]*	*39.9 [36.2*–*43.6]*
*Cocaine*	*22.7*	*31.1 [30.0*–*32.1]*	*37.0 [32.4*–*41.6]*	*10.6*	*14.9 [14.4*–*15.3]*	*40.0 [35.5*–*44.5]*
Sedatives	9.9	15.6 [14.7–16.6]	58.4 [48.8–68.0]	18.7	30.0 [29.4–30.7]	60.7 [57.1–64.2]
*Benzodiazepines*	*6.7*	*11.4 [10.6*–*12.3]*	*69.6 [56.8*–*82.3]*	*15.7*	*25.6 [24.9*–*26.2]*	*62.6 [58.6*–*66.5]*
Psychotropics	14.6	21.2 [20.1–22.2]	44.7 [37.7–51.7]	18.0	26.0 [25.4–26.6]	*44.6* [41.2–48.0]
*Antidepressants*	*10.4*	*16.3* *[15.4*–*17.3]*	*57.1* *[47.9*–*66.3]*	*10.3*	*16.4* *[15.9*–*17.0]*	*60.2* *[55.0*–*65.4]*
*Antipsychotics*	*1.9*	*3.2* *[2.7*–*3.7]*	*68.9* *[43.0*–*94.7]*	*3.2*	*5.3* *[5.0*–*5.6]*	*65.1* *[54.7*–*75.5]*
*Stimulants*	*3.2*	*3.9* *[3.4*–*4.4]*	*19.9* *[5.3*–*34.6]*	*6.3*	*7.9* *[7.5*–*8.3]*	*24.4* *[18.6*–*30.2]*
Other specified	6.9	8.8 [8.1–9.5]	26.6 [16.8–36.3]	7.6	10.9 [10.5–11.3]	43.5 [37.8–49.2]
Unspecified	50.3	35.4 [34.3–36.5]	−29.6 [−31.7 – −27.4]	49.7	34.1 [33.4–34.7]	−31.4 [−32.7 – −30.2]
>1 Major drug class^e^	18.0	28.6 [27.5–29.8]	58.6 [52.2–64.9]	26.9	42.8 [42.1–43.5]	58.9 [56.3–61.5]

^a^Data from the Multiple Cause of Death files

^b^Proportions from death certificate reports

^c^Adjusted proportions are average predicted values from probit models, where at least one specific drug is assumed to be mentioned for all poisoning deaths (SPECIFY = 1). Models also control for: sex, race (black, other), Hispanic, currently married, education (high school dropout, high school graduate, some college, college graduate), age (≤20, 21–30, 31–40, 41–50, 51–60, 61–70, 71–80, >80), day of the week of death, and census region

^d^% Difference between adjusted and reported proportions (calculated using more precise proportions than the rounded percentages displayed on the table)

^e^Two or more of the drug types: opioid analgesics, other narcotics, sedatives, psychotropics, or other specified drugs

The adjustment procedures significantly raised the predicted frequency of all specific drug proportions, implying that death certificates understated most types of drug involvement. For example, the adjusted proportion of opioid analgesic mentions in 2012 was 54.3 % (95 % CI, 53.6 %–55 %), or 40.8 % (95 % CI, 39 %–42.5 %) higher than the reported 38.6 %. (Percentage differences vary slightly on the table due to rounding. In the example just shown, adjusted and reported proportions were actually 54.296 % and 38.569 %, and the difference between them was 40.776 %).

Adjusted proportions for narcotics, psychotropics, other specified drugs, and combination drug use exceeded reported shares by 34 % to 46 %, while those for unspecified drugs fell 31 %. The increase was a particularly large, at 60.7 % (95 % CI, 57.1 %–64.2 %) for sedatives – mostly due to the 62.6 % (95 % CI, 58.6 %–66.5 %) growth for benzodiazepines – and also for antipsychotics and antidepressants, where adjusted proportions exceeded reported values by 65.1 % (95 % CI, 54.7 %–75.5 %) and 60.2 % (95 % CI, 55 %–65.4 %). The adjustment procedure typically had similar or slightly smaller percentage effects on drug shares in 1999.

An alternative set of adjusted proportions was obtained from models that contained additional controls for the manner of death (accidental or intentional, with undetermined intent/homicides as the reference group). This specification has the potential advantage of increased predictive power, but will be problematic if classification of the manner of death depends on drug specification rates, as was suggested above. In practice, the adjusted shares were virtually identical to those obtained from the main model (Additional file 3).

Table 4 details adjusted proportions and percentage differences compared to death certificate reports in 1999 and 2012 for subsamples stratified by whether the fatal drug poisoning was classified as accidental, intentional, or of undetermined intent. Results for homicides are not reported since these accounted for just 0.2 % of drug mortality. (Additional file 4 shows reported proportions). Patterns of drug involvement vary substantially by manner of death, with narcotics – particularly non-analgesics – being much more commonly involved for accidental fatalities and those of undetermined intent; whereas sedatives, psychotropics, and other specified and unspecified drugs played bigger roles for intentional drug deaths. However, there were no clear patterns of percentage differences in adjusted versus reported proportions across years or manners of death.

**Table 4:** Adjusted drug involvement by manner of death and change vs. reported proportion^a^

Drug category	% [95 % CI]					
	Accidental		Intentional		Undetermined intent	
	Adjusted^b^	% Difference^c^	Adjusted^b^	% Difference^c^	Adjusted^b^	% Difference^c^
1999						
Narcotics	86.9 [86.0–87.7]	29.9 [28.6–31.2]	36.2 [33.3–39.2]	52.2 [40.1–64.9]	81.0 [78.6–83.4]	18.8 [15.4–22.3]
Opioid analg.	34.6 [33.2–36.0]	33.1 [27.7–38.5]	28.9 [26.1–31.7]	52.1 [37.2–66.9]	25.7 [23.0–28.4]	23.1 [10.2–35.9]
Other narcotics	68.6 [67.4–69.9]	36.0 [33.5–38.4]	9.8 [7.9–11.6]	62.9 [32.0–93.7]	63.1 [60.3–65.8]	19.8 [14.6–25.0]
Sedatives	13.3 [12.2–14.4]	67.6 [53.6–81.7]	30.8 [28.0–33.7]	62.0 [47.0–77.1]	10.7 [8.5–12.9]	56.8 [24.5–89.2]
Psychotropics	15.9 [14.7–17.0]	46.7 [36.2–57.2]	45.0 [42.0–48.0]	55.7 [45.3–66.1]	18.8 [16.2–21.3]	40.2 [21.0–59.4]
Other specified	6.9 [6.2–7.6]	17.5 [5.8–29.1]	18.7 [16.3–21.1]	45.4 [26.9–64.0]	5.9 [4.3–7.6]	48.2 [7.2–89.1]
Unspecified	33.1 [31.8–34.4]	−32.6 [−29.9 – −35.2]	55.0 [52.1–57.9]	−16.4 [−12.0 – −20.9]	25.0 [22.4–27.5]	−31.3 [−24.3 – −38.4]
>1 Drug class^d^	30.1 [28.7–31.5]	57.8 [50.4–65.3]	33.6 [30.5–36.7]	92.9 [75.2–110.6]	22.6 [19.8–25.4]	59.5 [39.8–79.2]
2012						
Narcotics	87.4 [86.9–87.8]	32.9 [32.2–33.6]	44.4 [42.5–46.3]	38.7 [32.8–44.6]	76.9 [74.7–79.1]	35.8 [32.0–39.6]
Opioid analg.	56.5 [55.8–57.3]	41.4 [39.6–43.3]	40.0 [38.1–41.9]	39.6 [33.1–46.2]	57.7 [55.1–60.3]	38.5 [32.3–44.8]
Other narcotics	43.9 [43.2–44.6]	36.3 [34.0–38.5]	6.2 [5.2–7.1]	40.3 [18.7–62.0]	30.8 [28.4–33.1]	39.0 [28.3–49.6]
Sedatives	29.7 [29.0–30.4]	64.8 [60.7–68.9]	36.2 [34.3–38.0]	48.6 [40.9–56.2]	24.6 [22.2–27.0]	56.4 [41.1–71.7]
Psychotropics	23.6 [23.0–24.3]	44.8 [40.8–48.9]	41.4 [39.5–43.3]	46.9 [40.2–53.6]	27.0 [24.6–29.5]	50.9 [37.3–64.6]
Other specified	8.2 [7.8–8.7]	51.8 [43.9–59.8]	28.8 [27.1–30.5]	33.4 [25.5–41.3]	8.9 [7.3–10.5]	46.6 [20.2–73.0]
Unspecified	32.2 [31.5–32.9]	−33.3 [−31.9 – −34.8]	48.6 [46.7–50.4]	−18.2 [−15.1 – −21.3]	30.6 [28.2–32.9]	−35.4 [−30.5 – −40.4]
>1 Drug class^d^	43.2 [42.4–44.0]	60.2 [57.3–63.1]	43.3 [41.3–45.2]	56.6 [49.7–63.6]	39.2 [36.5–41.9]	53.2 [42.8–63.7]

^a^Data from the Multiple Cause of Death files

^b^Adjusted proportions are average predicted values from probit models, where at least one specific drug is assumed to be mentioned for all poisoning deaths (SPECIFY = 1). Models also control for: sex, race (black, other), Hispanic, currently married, education (high school dropout, high school graduate, some college, college graduate), age (≤20, 21–30, 31–40, 41–50, 51–60, 61–70, 71–80, >80), day of the week of death, and census region

^c^% Difference shows the percentage difference between adjusted proportions and those obtained directly from mentions on death certificates

^d^Two or more of the drug types: opioid analgesics, other narcotics, sedatives, psychotropics, or other specified drugs

Adjusted shares and percentage differences versus reported drug involvement are shown separately for 16–59 and ≥60 year olds in Table 5. (Additional file 5 provides corresponding reported proportions). Information for persons younger than 16 is not displayed since they accounted for less than 0.6 % of drug fatalities. There were more than eight times as many drug deaths among 16–59 year olds (15,484 in 1999 and 37,171 in 2012) as ≥60 year olds (1257 in 1999 and 4182 in 2012), generally resulting in more precise adjusted proportions for the younger group.

**Table 5:** Adjusted drug involvement by age group and change vs. reported proportion^a^

Drug category	% [95 % ci]			
	16–59 year olds		≥60 year olds	
	Adjusted^b^	% Difference^c^	Adjusted^b^	% Difference^c^
1999				
Narcotics	81.1 [80.2–82.0]	31.4 [29.9–32.8]	36.5 [32.3–40.7]	39.5 [23.3–55.7]
Opioid analgesics	32.0 [30.8–33.2]	30.4 [25.6–35.1]	23.4 [19.5–27.3]	42.0 [18.1–65.9]
Other narcotics	61.8 [60.7–62.9]	37.3 [34.8–39.8]	16.6 [13.6–19.6]	40.7 [15.2–66.2]
Sedatives	15.2 [14.3–16.2]	60.8 [50.5–71.2]	20.8 [17.0–24.6]	39.2 [13.8–64.6]
Psychotropics	21.3 [20.3–22.4]	44.4 [37.2–51.7]	20.3 [16.5–24.1]	52.8 [24.1–81.5]
Other specified	6.7 [6.1–7.4]	38.3 [24.8–51.9]	32.3 [28.2–36.4]	3.8 [−9.2–16.9]
Unspecified	35.4 [34.3–36.6]	−30.5 [−28.2 – −32.7]	36.7 [32.5–40.8]	−15.9 [−6.4 – −25.3]
>1 Major drug class^d^	29.6 [28.4–30.8]	58.2 [51.8–64.7]	18.3 [14.6–22.1]	63.5 [30.0–97.0]
2012				
Narcotics	83.4 [82.9–84.0]	34.2 [33.4–35.0]	64.0 [62.1–66.0]	35.2 [31.1–39.4]
Opioid analgesics	54.9 [54.2–55.6]	41.0 [39.2–42.9]	48.9 [46.7–51.1]	39.2 [33.1–45.3]
Other narcotics	40.6 [39.9–41.3]	38.2 [35.9–40.5]	20.2 [18.6–21.8]	34.4 [23.8–45.0]
Sedatives	30.2 [29.6–30.9]	60.7 [57.0–64.4]	28.8 [26.7–30.8]	60.5 [49.1–71.8]
Psychotropics	25.9 [25.2–26.5]	44.8 [41.1–48.4]	27.5 [25.6–29.5]	44.0 [33.8–54.3]
Other specified	9.6 [9.2–10.1]	48.6 [41.8–55.5]	22.5 [20.8–24.2]	28.9 [19.1–38.8]
Unspecified	34.1 [33.4–34.7]	−31.9 [−30.5 – −33.2]	34.5 [32.5–36.5]	−26.7 [−22.5 – −30.9]
>1 Major drug class^d^	43.6 [42.9–44.4]	58.8 [56.1–61.5]	36.4 [34.2–38.5]	60.8 [51.3–70.3]

^a^Data from the Multiple Cause of Death files

^b^Adjusted proportions are average predicted values from probit models, where at least one specific drug is assumed to be mentioned for all poisoning deaths (SPECIFY = 1). Models also control for: sex, race (black, other), Hispanic, currently married, education (high school dropout, high school graduate, some college, college graduate), age (≤20, 21–30, 31–40, 41–50, 51–60, 61–70, 71–80, >80), day of the week of death, and census region

^c^% Difference shows the percentage difference between adjusted proportions and those obtained directly from mentions on death certificates

^d^Two or more of the drug types: opioid analgesics, other narcotics, sedatives, psychotropics, or other specified drugs

Compared to drug deaths involving persons 60 and over, those among 16–59 year olds more often involved narcotics of all kinds and combination drug use, with other specified drugs less often implicated but with no clear patterns for sedatives, psychotropics or unspecified drugs. Reported proportions understate drug involvement by similar amounts for the two age groups with two exceptions: there was a particularly large downwards bias in the share of other drugs for 16–59 year olds in 1999 and 2012, as well as for sedatives in 1999.

### Number of deaths

The first and third columns of Table 6 show estimates for the number of drug poisoning deaths involving major categories of drugs, based on the adjusted proportions detailed in Table 3. The second and fourth columns indicate how these differ from the corresponding numbers reported on death certificates. To illustrate, 22,534 drug deaths were estimated to involve opioid analgesics in 2012, calculated as the adjusted proportion (54.296 %) times the total number of drug poisoning deaths (41,502). This number is 6527 above the 16,007 death certificates mentioning this type of drug. The calculations use more significant digits than reported on the tables, so that the small differences occur compared to calculations based on the rounded estimates.

**Table 6:** Adjusted number of drug poisoning deaths and change vs. reported numbers^a^

Drug category	# [95 % CI]			
	1999		2012	
	Deaths^b^	∆ vs. reported^c^	Deaths^b^	∆ vs. reported^c^
Narcotics	13,114 [12,962–13,265]	3192 [3040–3343]	33,830 [33,560–34,100]	8643 [8373–8913]
Opioid analgesics	5275 [5087–5463]	1245 [1057–1433]	22,534 [22,254–22,814]	6527 [6247–6807]
Other narcotics	9820 [9641–9999]	2683 [2504–2862]	15,933 [15,671–16,196]	4366 [4104–4629]
Sedatives	2633 [2473–2792]	971 [811–1130]	12,457 [12,185–12,729]	4703 [4431–4975]
Psychotropics	3568 [3395–3741]	1102 [929–1275]	10,798 [10,544–11,053]	3331 [3077–3586]
Other specified	1482 [1368–1596]	311 [197–425]	4528 [4348–4709]	1372 [1192–1553]
Unspecified	5930 [5786–6154]	−2507 [−2691 – −2323]	14,135 [13,874–14,396]	−6477 [−6738 – −6216]
>1 Major drug class^d^	4820 [4627–5013]	1780 [1587–1973]	17,670 [17,469–18,050]	6584 [6293–6874]

^a^Data from the Multiple Cause of Death files

^b^Number of drug poisoning deaths involving the specified drug, calculated as the product of the number of drug poisoning deaths in the year multiplied by the adjusted proportion obtained from probit models, where at least one specific drug is assumed to be mentioned for all poisoning deaths (SPECIFY = 1). Models also control for: sex, race (black, other), Hispanic, currently married, education (high school dropout, high school graduate, some college, college graduate), age (≤20, 21–30, 31–40, 41–50, 51–60, 61–70, 71–80, >80), day of the week of death, and census region. The calculations use more significant digits for adjusted proportions than are shown on Table 3, so that there may be small differences from those that would be obtained using rounded estimates

^c^Difference between adjusted number of deaths and unadjusted number based on death certificate reports

^d^Two or more of the drug types: opioid analgesics, other narcotics, sedatives, psychotropics, or other specified drugs.

The adjustments raise the number of deaths involving all specific drug categories, while reducing the role of unspecified drugs. Larger increases were observed in 2012 than in 1999, as expected given the dramatic growth in total drug poisoning mortality, and these differences show the extent to which death certificates inaccurately indicate trends in drug involvement. The biggest absolute disparities were for opioid analgesics: 6527 deaths (95 % CI 6247–6807) in 2012 versus 1245 (95 % CI 1057–1433) in 1999 and combination drug use 6584 deaths (95 % CI 6293–6874) versus 1780 (95 % CI 1587–1973). However, the greatest relative differences were for sedatives and psychotropic medications. (Additional file 6 shows results for drug subcategories).

## Discussion

The information currently provided on death certificates is inadequate for understanding the nature of the drug poisoning epidemic. Particularly problematic is the frequency with which no specific drug is identified [20]. Since this is more common when death certificates are completed by coroners rather than medical examiners and in states without centralized oversight by a Chief Medical Examiner [21], additional training and standardization in states with low drug specification rates may be helpful. Others have also recommended including more detail on the drugs involved in poisoning deaths, toxicology levels of opioids, whether or not these were prescribed to decedent, and more detailed opioid toxicology ICD categories [22, 23].

Until better information becomes available, the predictive adjustment methods developed here can provide more accurate estimates of specific drug involvement. The adjustments are substantial, as death certificates understated mentions of major classes of drugs by 27 % to 58 % in 1999 and by 34 % to 61 % in 2012 and combination drug use by 59 % in both years, with even larger changes for some subcategories of drugs (e.g. antipsychotics and benzodiazepines). Underlying causes of death are also inaccurately recorded because they are classified based upon reported drug mentions. For example, narcotics was classified as the UCD in 46.4 % of drug poisoning cases in 1999 and 37.6 % in 2012, compared to adjusted rates of 59.6 % and 48 % respectively, using the procedures to described above (Additional file 7).

The adjustment procedures work well, but not perfectly. As evidence of this, reported proportions of exclusive unspecified drug mentions were 21.9 % in 1999 and 24.2 % in 2012, while the adjusted shares were 4.3 % and 3.7 %. Completely successful adjustment would reduce the adjusted proportion to zero, indicating that 80 % and 85 % (rather than 100 %) of such mentions were eliminated. Adjusted proportions calculated under the assumption that drug types were never specified on the death certificates (by predicting probabilities after setting *SPECIFY* = 0) were 96.4 % in 1999 and 96.5 % in 2012, which is close to the 100 % adjusted share occurring with perfect prediction. These favorable results occur even though goodness-of-fit, as measured by the pseudo-R^2^, which ranges between 0.06 and 0.33, implies a limited ability to predict drug use in individual cases. This does not necessarily indicate inaccuracy in the overall estimates of adjusted proportions, but could be problematic if unobserved determinants of drug involvement were spuriously correlated with county-level specification rates.

Better understanding is also needed of the situations under which the unspecified drug category (T50.9) is reported on death certificates. The procedures developed here adjust for cases where unspecified drugs are exclusively mentioned (i.e., when death certificates report a drug poisoning without identifying any of the drugs involved). However, in many cases there are mentions of both specified and unspecified drugs. These sometimes occur when “multidrug toxicity” or something similar, is listed on one part of the death certificate but with more specific information, such as heroin use, reported on another part (source: personal communication with Robert Anderson, Chief of the Mortality Statistics Branch of the National Center for Health Statistics, October 2, and October 6, 2015).

In 1999, 16,849 US residents died of drug poisoning. By 2012, the number had risen to 41,502. Mentions of opioid analgesic almost quadrupled, from 4030 to 16,007, but the adjusted estimates indicate an even larger increase in opioid involvement from 5275 deaths (95 % CI 5087–5463) in 1999 to 22,534 (95 % CI 22,254–22,814) in 2012. These figures imply that these drugs were mentioned in 54.3 % (95 % CI, 53.6 %–55 %) of fatal drug poisonings in the later year. Such results justify efforts to reduce the negative consequences of prescription opioid use, including establishing prescription drug monitoring programs; restrictions on the dispensing of oxycodone and other controlled substances from pain clinics and online pharmacies; and development of abuse-deterrent drug formulations [24–28]. These endeavors have been somewhat successful. Drug poisoning deaths in Florida decreased 17 % between 2010 and 2013, with a 52 % decline in fatalities involving oxycodone following aggressive efforts to reverse the proliferation of pain clinics, prohibit the dispensing of schedule II or III drugs from physician offices, and other measures [29]. Deaths involving methadone peaked in 2007 and then declined along with a fall in the amount of methadone distributed nationally [5]. However, the accomplishments are incomplete. After Florida’s crackdown, some pain clinic owners moved out of the state or found ways to circumvent the laws, and it is questionable whether prescription drug monitoring programs have reduced drug poisoning deaths [24, 29, 30]. Most notably, fatalities involving heroin have doubled since 2004, raising the possibility of the substitution of heroin for prescription opioids, although evidence on this is conflicting [7, 31, 32].

Nor should attention be limited to the risks of prescription opioids. Deaths involving sedatives, especially benzodiazepines, have grown even faster in percentage terms, and involvement of these drugs and of psychotropic medications is particularly severely understated on death certificates. Attention has been paid to the role of non-opioids and to combination drug use, but a better understanding is needed since opioids and benzodiazepines are often combined, with greater resulting health risks than the use of either alone [2, 8, 33, 34]. In a more general sense, comprehensive efforts to reduce drug fatalities should account for potential therapeutic benefits and substitution between drugs, the frequency of combination drug use, and the heterogeneity in levels and growth of drug mortality across geographic areas and demographic groups [2, 35, 36].

The results of this analysis are subject to additional caveats. First, the drugs identified as contributing to fatal drug poisonings may sometimes be reported inaccurately. For instance, heroin use may sometimes be misattributed to morphine or codeine, because heroin metabolizes into morphine and codeine may be detected as an impurity in either morphine or heroin [37]. Second, some deaths could be misclassified as being due to non-drug causes, and therefore be excluded from the analysis, while others that are defined as drug poisonings may actually primarily result from non-drug causes. Third, the adjustment procedures do not fully correct for cases where death certificates mention both unspecified and specified types of drugs. Fourth, information on specific drug involvement was not available on death certificates, and so not examined here, for the small number of cases (732 in 1999 and 637 in 2012) where the underlying cause was classified as a mental or behavior disorder due to drug use (ICD-10 codes F11 through F16). Fifth, while population-wide adjusted proportions are provided, it would be useful to obtain estimates for subcategories. However, the precision of such estimates will be limited for groups where the number of deaths is small, and this is likely to make it difficult to construct age-adjusted proportions using finely defined age categories. Finally, future research should examine whether these adjustments change the estimated consequences of policies potentially affecting drug deaths, such as those related to prescription drug monitoring programs, internet pharmacies, and medical cannabis laws [29, 38, 39].

## Conclusions

Death certificates substantially understate the frequency of involvement of opioid analgesics, sedatives, psychotropics, and combinations of drugs in fatal drug poisonings. Adjustment procedures that account for cases where only unspecified drugs are reported on death certificates provide more accurate and informative estimates.

## Additional files

Additional file 1: **Percent of drug poisoning deaths with at least one drug specified**^**a**^**.** (DOCX 39 kb)

Additional file 2: **Actual and predicted drug mentions**^**a**^**.** (DOCX 35 kb)

Additional file 3: **Adjusted drug involvement, with and without controls for manner of death**^**a**^**.** (DOCX 39 kb)

Additional file 4: **Reported drug involvement by manner of death**^**a**^**.** (DOCX 36 kb)

Additional file 5: **Reported drug involvement by age**^**a**^**.** (DOCX 37 kb)

Additional file 6: **Adjusted number of drug poisoning deaths and percent change vs. reported numbers for drug subcategories**^**a**^**.** (DOCX 41 kb)

Additional file 7: **Reported and adjusted underlying cause of death shares**^**a**^**.** (DOCX 37 kb)
