# Supplementary material for: Drug poisoning deaths in the United States, 1999–2012: a statistical adjustment analysis
Source: Popul Health Metr. 2016 Jan 15;14:2. doi: 10.1186/s12963-016-0071-7 (PMC4714527; doi:10.1186/s12963-016-0071-7)
Supplement: Supplementary file 1 — Percent of drug poisoning deaths with at least one drug specifieda. (DOCX 39 kb) [file 12963_2016_71_MOESM1_ESM.docx]

Additional File 1: Percent of drug poisoning deaths with at least one drug specified^a^

| State | 1999 | 2012 | State | 1999 | 2012 |
| --- | --- | --- | --- | --- | --- |
| *Alabama*^b^ | *55.0* | *41.1* | *Montana* | *68.3* | *60.2* |
| **Alaska^c^** | **93.5** | **89.9** | *Nebraska* | *64.1* | *68.5* |
| Arizona | 75.1 | 67.9 | **Nevada** | **93.8** | **96.3** |
| Arkansas | 69.0 | 69.7 | **New Hampshire** | **98.1** | **97.1** |
| California | 80.2 | 72.0 | New Jersey | 80.1 | 64.2 |
| Colorado | 84.2 | 69.3 | New Mexico | 93.6 | 83.5 |
| Connecticut | 74.8 | 62.9 | **New York** | **93.7** | **94.6** |
| Delaware | 82.0 | 70.2 | North Carolina | 89.6 | 84.6 |
| District of Columbia | 95.8 | 87.0 | *North Dakota* | *66.7* | *61.9* |
| Florida | 71.9 | 71.5 | Ohio | 68.3 | 78.2 |
| Georgia | 69.3 | 73.5 | **Oklahoma** | **91.6** | **94.9** |
| Hawaii | 85.0 | 83.9 | Oregon | 85.2 | 95.7 |
| *Idaho* | *65.6* | *46.4* | *Pennsylvania* | *50.5* | *46.6* |
| Illinois | 91.5 | 87.8 | **Rhode Island** | **96.6** | **95.4** |
| *Indiana* | *58.6* | *47.7* | South Carolina | 74.1 | 58.4 |
| Iowa | 88.7 | 94.6 | South Dakota | 82.4 | 97.6 |
| Kansas | 71.9 | 72.6 | Tennessee | 70.1 | 83.9 |
| Kentucky | 49.2 | 71.2 | Texas | 69.1 | 74.4 |
| *Louisiana* | *40.4* | *41.9* | **Utah** | **97.1** | 94.7 |
| Maine | 79.1 | 88.0 | Vermont | 86.2 | 98.6 |
| **Maryland** | **91.9** | **97.5** | **Virginia** | **89.9** | **98.0** |
| **Massachusetts** | **94.7** | **98.1** | **Washington** | **94.6** | **95.5** |
| *Michigan* | *52.2* | *64.1* | West Virginia | 89.3 | 98.6 |
| Minnesota | 83.8 | 83.4 | Wisconsin | 86.3 | 87.1 |
| *Mississippi* | *46.0* | *45.2* | Wyoming | 75.0 | 63.3 |
| Missouri | 82.6 | 76.4 |  |  |  |

^a^ Data from the Multiple Cause of Death files. Table shows shares of death in given year where at least one specific drug is mentioned on death certificate.

^b^ States in italics are low diagnosis states, defined as those with at least one drug specified for fewer than 68.8% of drug poisoning deaths in both 1999 and 2012.

^c^ States in bold are high diagnosis states, defined as those with at least one drug specified for more than 89.6% of drug poisoning deaths in both 1999 and 2012.
