# Supplementary material for: Drug poisoning deaths in the United States, 1999–2012: a statistical adjustment analysis
Source: Popul Health Metr. 2016 Jan 15;14:2. doi: 10.1186/s12963-016-0071-7 (PMC4714527; doi:10.1186/s12963-016-0071-7)
Supplement: Supplementary file 2 — Actual and predicted drug mentionsa. (DOCX 35 kb) [file 12963_2016_71_MOESM2_ESM.docx]

| Additional File 2: Actual and predicted drug mentions^a^ | | | | |
| --- | --- | --- | --- | --- |
| Drug category | % | | | |
|  | 1999 | | 2012 | |
|  | Actual^b^ | Predicted^c^ | Actual^b^ | Predicted^c^ |
| Narcotics | 58.9 | 59.0 | 60.7 | 60.7 |
| Opioid analgesics | 23.9 | 23.9 | 38.6 | 38.6 |
| *Methadone* | *4.7* | *4.8* | *9.5* | *9.5* |
| *Other opioid analgesics* | *19.9* | *19.9* | *31.4* | *31.4* |
| Other narcotics | 42.4 | 42.4 | 27.9 | 27.9 |
| *Heroin* | *11.6* | *11.6* | *14.3* | *14.3* |
| *Cocaine* | *22.7* | *22.6* | *10.6* | *10.7* |
| Sedatives | 9.9 | 9.9 | 18.7 | 18.7 |
| *Benzodiazepines* | *6.7* | *6.7* | *15.7* | *15.7* |
| Psychotropics | 14.6 | 14.6 | 18.0 | 18.0 |
| *Antidepressants* | *10.4* | *10.4* | *10.3* | *10.2* |
| *Antipsychotics* | *1.9* | *1.9* | *3.2* | *3.2* |
| *Stimulants* | *3.2* | *3.3* | *6.3* | *6.4* |
| Other specified | 6.9 | 6.9 | 7.6 | 7.6 |
| Unspecified | 50.3 | 50.2 | 49.7 | 49.6 |
| >1 Major drug class^d^ | 18.0 | 18.0 | 26.9 | 27.0 |

^a^ Data from the Multiple Cause of Death files.

^b^ Involvement rates from death certificate reports.

^c^ Average predicted values from probit models that control for actual state rates at which at least one specific drug is assumed to be mentioned for all poisoning deaths (SPECIFY), as well as: sex, race (black, other), Hispanic, currently married, education (high school dropout, high school graduate, some college, college graduate), age (≤20, 21-30, 31-40, 41-50, 51-60, 61-70, 71-80, >80), day of the week of death, and census region.

^d^ Two or more of the drug types: opioid analgesics, other narcotics, sedatives, psychotropics, or other specified drugs.
