# Supplementary material for: Drug poisoning deaths in the United States, 1999–2012: a statistical adjustment analysis
Source: Popul Health Metr. 2016 Jan 15;14:2. doi: 10.1186/s12963-016-0071-7 (PMC4714527; doi:10.1186/s12963-016-0071-7)
Supplement: Supplementary file 3 — Adjusted drug involvement, with and without controls for manner of deatha. (DOCX 39 kb) [file 12963_2016_71_MOESM3_ESM.docx]

| Additional File 3: Adjusted drug involvement, with and without controls for manner of death^a^ | | | | |
| --- | --- | --- | --- | --- |
| Drug category | 1999 | | 2012 | |
|  | Adjusted w/o manner^b^ | Adjusted with manner^c^ | Adjusted w/o manner^b^ | Adjusted with manner^c^ |
| Narcotics | 77.8  [76.9-78.7] | 77.7  [76.8-78.5] | 81.5  [80.9-82.2] | 81.6  [81.1-82.1] |
| Opioid analgesics | 31.3  [30.2-32.4] | 31.4  [30.3-32.5] | 54.3  [53.6-55.0] | 54.4  [53.7-55.0] |
| Other narcotics | 58.3  [57.2-59.3] | 57.8  [56.8-58.8] | 38.4  [37.8-39.0] | 38.5  [37.8-39.1] |
| *Heroin* | *14.1*  *[13.3-14.9]* | *14.2*  *[13.4-15.0]* | *20.0*  *[19.4-20.5]* | *20.0*  *[19.5-20.5]* |
| *Cocaine* | *31.1*  *[30.0-32.1]* | *31.1*  *[30.1-32.1]* | *14.9*  *[14.4-15.3]* | *14.9*  *[14.4-15.4]* |
| Sedatives | 15.6  [14.7-16.6] | 15.7  [14.8-16.6] | 30.0  [29.4-30.7] | 30.0  [29.4-30.7] |
| Psychotropics | 21.2  [20.1-22.2] | 21.1  [20.1-22.1] | 26.0  [25.4-26.6] | 25.9  [25.3-26.6] |
| *Antidepressants* | *16.3*  *[15.4-17.3]* | *16.2*  *[15.3-17.1]* | *16.4*  *[15.9-17.0]* | *16.4*  *[15.8-16.9]* |
| *Antipsychotics* | *3.2*  *[2.7-3.7]* | *3.2*  *[2.7-3.7]* | *5.3*  *[5.0-5.6]* | *5.3*  *[4.9-5.6]* |
| *Stimulants* | *3.9*  *[3.4-4.4]* | *3.9*  *[3.5-4.4]* | *7.9*  *[7.5-8.3]* | *7.9*  *[7.6-8.3]* |
| Other specified | 8.8  [8.1-9.5] | 8.8  [8.1-9.4] | 10.9  [10.5-11.3] | 10.8  [10.4-11.2] |
| Unspecified | 35.4  [34.3-36.5] | 35.6  [34.6-36.7] | 34.1  [33.4-34.7] | 34.0  [33.4-34.6] |
| >1 Major drug class^d^ | 28.6  [27.5-29.8] | 28.7  [27.6-29.8] | 42.8  [42.1-43.5] | 42.8  [42.1-43.5] |

^a^ Data from the Multiple Cause of Death files.

^b^ Adjusted proportions are average predicted values from probit models, where at least one specific drug is assumed to be mentioned for all poisoning deaths (SPECIFY =1). Models also control for: sex, race (black, other), Hispanic, currently married, education (high school dropout, high school graduate, some college, college graduate), age (≤20, 21-30, 31-40, 41-50, 51-60, 61-70, 71-80, >80), day of the week of death, and census region.

^c^ Adjusted proportions are average predicted values from probit models, where at least one specific drug is assumed to be mentioned for all poisoning deaths (SPECIFY =1). Models also control for: sex, race, Hispanic, currently married, education, age, day of the week of death, census region, and manner of death (accidental, intentional, undetermined intent/homicide).

^d^ Two or more of the drug types: opioid analgesics, other narcotics, sedatives, psychotropics, or other specified drugs.
