# Supplementary material for: Drug poisoning deaths in the United States, 1999–2012: a statistical adjustment analysis
Source: Popul Health Metr. 2016 Jan 15;14:2. doi: 10.1186/s12963-016-0071-7 (PMC4714527; doi:10.1186/s12963-016-0071-7)
Supplement: Supplementary file 4 — Reported drug involvement by manner of deatha. (DOCX 36 kb) [file 12963_2016_71_MOESM4_ESM.docx]

| Additional File 4: Reported drug involvement by manner of death^a^ | | | | | | | |
| --- | --- | --- | --- | --- | --- | --- | --- |
| Drug category | % | | | | | | |
|  | Accidental | | | Intentional | | Undetermined intent | |
|  | 1999 | 2012 | | 1999 | 2012 | 1999 | 2012 |
| Narcotics | 66.9 | | 65.8 | 23.8 | 32.0 | 68.2 | 56.6 |
| Opioid analg. | 26.0 | | 40.0 | 19.0 | 28.7 | 20.9 | 41.7 |
| Other narcotics | 50.5 | | 32.2 | 6.0 | 4.4 | 52.6 | 22.1 |
| Sedatives | 7.9 | | 18.0 | 19.0 | 24.4 | 6.8 | 15.7 |
| Psychotropics | 10.8 | | 16.3 | 28.9 | 28.2 | 13.4 | 17.9 |
| Other specified | 5.9 | | 5.4 | 12.9 | 21.6 | 4.0 | 6.1 |
| Unspecified | 49.0 | | 48.3 | 65.8 | 59.4 | 36.4 | 47.4 |
| >1 Drug class^b^ | 19.1 | | 27.0 | 17.4 | 27.6 | 14.2 | 25.6 |

^a^ Data from the Multiple Cause of Death files.

^b^ Two or more of the drug types: opioid analgesics, other narcotics, sedatives, psychotropics, or other specified drugs.
