# Supplementary material for: Drug poisoning deaths in the United States, 1999–2012: a statistical adjustment analysis
Source: Popul Health Metr. 2016 Jan 15;14:2. doi: 10.1186/s12963-016-0071-7 (PMC4714527; doi:10.1186/s12963-016-0071-7)
Supplement: Supplementary file 5 — Reported drug involvement by agea. (DOCX 37 kb) [file 12963_2016_71_MOESM5_ESM.docx]

| Additional File 5: Reported drug involvement by age^a^ | | | | |
| --- | --- | --- | --- | --- |
| Drug category | % | | | |
|  | 16-59 year olds | | ≥60 year olds | |
|  | 1999 | 2012 | 1999 | 2012 |
| Narcotics | 61.7 | 62.2 | 26.2 | 47.4 |
| Opioid analgesics | 24.6 | 38.9 | 16.5 | 35.1 |
| Other narcotics | 45.0 | 29.4 | 11.8 | 15.1 |
| Sedatives | 9.5 | 18.8 | 15.0 | 17.9 |
| Psychotropics | 14.8 | 17.9 | 13.3 | 19.1 |
| Other specified | 4.9 | 6.5 | 31.1 | 17.4 |
| Unspecified | 51.0 | 50.0 | 43.6 | 47.1 |
| >1 Drug class^b^ | 18.7 | 27.5 | 11.2 | 22.6 |

^a^ Data from the Multiple Cause of Death files.

^b^ Two or more of the drug types: opioid analgesics, other narcotics, sedatives, psychotropics, or other specified drugs.
