# Supplementary material for: Drug poisoning deaths in the United States, 1999–2012: a statistical adjustment analysis
Source: Popul Health Metr. 2016 Jan 15;14:2. doi: 10.1186/s12963-016-0071-7 (PMC4714527; doi:10.1186/s12963-016-0071-7)
Supplement: Supplementary file 6 — Adjusted number of drug poisoning deaths and percent change vs. reported numbers for drug subcategoriesa. (DOCX 41 kb) [file 12963_2016_71_MOESM6_ESM.docx]

| Additional File 6: Adjusted number of drug poisoning deaths and percent change vs. reported numbers for drug subcategories^a^ | | | | |
| --- | --- | --- | --- | --- |
| Drug category | # [95% CI] | | | |
|  | 1999 | | 2012 | |
|  | # Deaths^b^ | Δ vs. reported^c^ | # Deaths^b^ | Δ vs. reported^c^ |
| Opioid analgesics | 5,275  [5,087-5,463] | 1,245  [1,057-1,433] | 22,534  [22,254-22,814] | 6,527  [6,247-6,807] |
| *Methadone* | *1,320*  *[1,199-1442]* | *536*  *[415-658]* | *5,624*  *[5,423-5,825]* | *1,692*  *[1,491-1,893]* |
| *Other opioid analg.* | *4,235*  *[4,058-4,411]* | *875*  *[698-1,051]* | *18,619*  *[18,335-18,903]* | *5,607*  *[5,323-5,891]* |
| Other narcotics | 9,820  [9,641-9,999] | 2,683  [2,504-2,862] | 15,933  [15,671-16,196] | 4,366  [4,104-4,629] |
| *Heroin* | *2,370*  *[2,238-2,502]* | *410*  *[278-542]* | *8,288*  *[8,067-8,509]* | *2,363*  *[2,142-2,584]* |
| *Cocaine* | *5,237*  *[5,062-5,413]* | *1,415*  *[1,240-1,591]* | *6,165*  *[5,967-6,363]* | *1,761*  *[1,563-1,959]* |
| Sedatives | 2,633  [2,473-2,792] | 971  [811-1,130] | 12,457  [12,185-12,729] | 4,703  [4,431-4,975] |
| *Benzodiazepines* | *1,925*  *[1,780-2,069]* | *790*  *[645-934]* | *10,605*  *[10,345-10,866]* | *4,081*  *[3,821-4,342]* |
| Psychotropics | 3,568  [3,395-3,741] | 1,102  [929-1,275] | 10,798  [10,544-11,053] | 3,331  [3,077-3,586] |
| *Antidepressants* | *2,748*  *[2,587-2,908]* | *999*  *[838-1,159]* | *6,822*  *[6,601-7,043]* | *2,563*  *[2,342-2,784]* |
| *Antipsychotics* | *542*  *[459-625]* | *221*  *[138-304]* | *2,200*  *[2,062-2,339]* | *867*  *[729-1,006]* |
| *Stimulants* | *656*  *[576-736]* | *109*  *[29-189]* | *3,277*  *[3,124-3,430]* | *642*  *[489-795]* |

^a^ Data from the Multiple Cause of Death files.

^b^ Number of drug poisoning deaths involving the specified drug or in the specified underlying cause of death category, estimated under the scenario where at least one specific drug is mentioned for all poisoning deaths (SPECIFY =1). Calculated as the product of the number of drug poisoning deaths in the year multiplied by the predicted predicted proportion involving the drug obtained from probit models, where at least one specific drug is assumed to be mentioned for all poisoning deaths (SPECIFY =1). Models also control for: sex, race (black, other), Hispanic, currently married, education (high school dropout, high school graduate, some college, college graduate), age (≤20, 21-30, 31-40, 41-50, 51-60, 61-70, 71-80, >80), day of the week of death, and census region.

^c^ Difference between adjusted number of deaths and unadjusted number based on death certificate reports.
