# Supplementary material for: Drug poisoning deaths in the United States, 1999–2012: a statistical adjustment analysis
Source: Popul Health Metr. 2016 Jan 15;14:2. doi: 10.1186/s12963-016-0071-7 (PMC4714527; doi:10.1186/s12963-016-0071-7)
Supplement: Supplementary file 7 — Reported and adjusted underlying cause of death sharesa. (DOCX 37 kb) [file 12963_2016_71_MOESM7_ESM.docx]

| Additional File 7: Reported and adjusted underlying cause of death shares^a^ | | | | | |  | |
| --- | --- | --- | --- | --- | --- | --- | --- |
| Underlying cause of death | % [95% CI] | | | | | | |
|  | 1999 | | | 2012 | | | |
|  | Reported^b^ | Adjusted^c^ | % Difference^d^ | Reported^b^ | Adjusted^c^ | | % Difference^d^ |
| Narcotics (X42, X62, Y12) | 46.4 | 59.6  [58.5-60.6] | 28.4  [26.1-30.8] | 37.6 | 48.0  [47.4-48.7] | | 27.8  [26.0-29.5] |
| Pain/psych/nerv (x40-41, x43,  x60-61, x63, y10-11,13) | 13.0 | 15.0  [14.1-15.9] | 15.6  [8.9-22.2] | 9.7 | 11.5  [11.1-11.9] | | 18.9  [14.4-23.4] |
| Other drug^e^ (x44, x64, y14) | 40.4 | 26.0  [25.0-27.0] | -35.7  [-38.1--33.2] | 52.5 | 40.7  [40.1-41.4] | | -22.5  [-23.7--21.2] |

^a^ Data from the Multiple Cause of Death files. ICD-10 underlying cause of death codes shown in parentheses.

^b^ Prevalences from death certificate reports.

^c^ Adjusted shares are average predicted values from probit models, where at least one specific drug is assumed to be mentioned for all poisoning deaths (SPECIFY =1). Models also control for: sex, race (black, other), Hispanic, currently married, education (high school dropout, high school graduate, some college, college graduate), age (≤20, 21-30, 31-40, 41-50, 51-60, 61-70, 71-80, >80), day of the week of death, and census region.

^d^ % Difference between adjusted and reported shares (calculated using more precise proportions than rounded percentages displayed on the table).

^e^ Two or more of the drug types: opioid analgesics, other narcotics, sedatives, psychotropics, or other specified drugs.
